# Supplementary material for: Splice-Junction-Based Mapping of Alternative Isoforms in the Human Proteome
Source: Cell Rep. Author manuscript; Available in PMC 2020 Jan 15. (PMC6961840; doi:10.1016/j.celrep.2019.11.026)

A

sp|O95299|NDUAA_HUMAN|ENSG00000130414|A5SS1|2177|chr2|240018639|240021412|−2|r194|T4
LLQYSDALEHLLTTGAVFLGSATSASAR q value: 0.0031207 Tr_novel:TRUE RefSeq_Novel:TRUE
Search result spec prec mz: 724.3863 Actual spec prec mz: 724.38629
Fragments matched per AA: 1.68 Proportion of top 20 peaks matched: 0.25

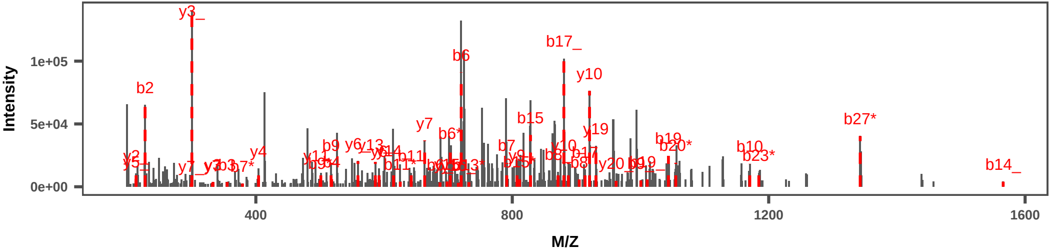

B

Scatterplot of predicted elution time
Fitting R2: 0.845
Novel peptide residual Z score: −3.14
Number of peptides: 906

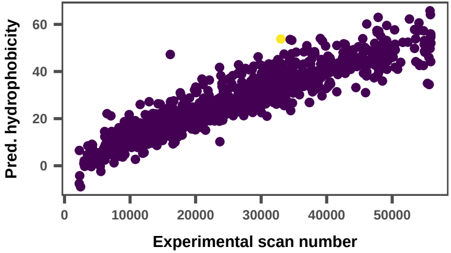

C

Distributions of residuals from best−fit line
of predicted RT vs Expt. scan number
Line: Z score of novel peptide
Z: −3.14

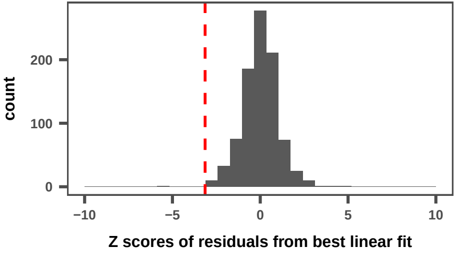

Supplement: 2 [file NIHMS1546469-supplement-2.zip › DF1/PXD010154/Liver/Liver_36_NDUFA10_LLQYSDALEHLLTTGAVFLGSATSASAR.pdf]
